# Parent-to-parent advice before the birth of a stillborn baby

**DOI:** 10.3389/fpsyt.2025.1736265

**Published:** 2025-12-18

**Authors:** Jennifer M. D. Kremkow, Jessica Lamberson

**Affiliations:** 1 Department of Communication Sciences and Disorders, Elmhurst University, Elmhurst, IL, United States; 2 Department of Nursing and Public Health, Elmhurst University, Elmhurst, IL, United States

**Keywords:** stillbirth, perinatal bereavement, loss, grief, parent experience

## Abstract

**Introduction:**

Stillbirth is a traumatic experience for parents who expect to welcome a living baby into their family, but find out during their pregnancy or during birth that their baby has died. Some stillbirth literature focuses on the lived experiences of parents such as memory making activities; however, few articles offer peer advice to newly bereaved parents.

**Methods:**

A self-administered online survey was utilized to collect parent-to-parent advice for newly bereaved parents. After data cleaning, 194 parent participant responses to the question “What should new loss parents know before the birth of their stillborn baby?” remained. Content analysis was used to analyze the responses.

**Results:**

Content analysis generated four main clusters respondents felt newly bereaved parents should know before the birth of their stillborn baby: (1) the birthing process, (2) the decisions about their baby, (3) memory making activities, and (4) the emotions before and after their baby’s birth.

**Discussion:**

This manuscript builds on limited previous literature by focusing on advice parents who experienced the birth of a stillborn baby would give to new loss parents experiencing stillbirth before going to the hospital. Advice from parent participants may be helpful for newly bereaved parents, healthcare and mental health providers, and organizations or non-profits supporting newly bereaved families.

## Introduction

Stillbirth is a traumatic experience in which parents and families unexpectedly and unwillingly find themselves. Instead of planning for a joyful, positive birth experience, parents whose baby is stillborn experience a range of emotions, from shock and disbelief, to guilt, grief, and isolation (1–3). Beyond individual feelings of grief, bereaved parents are also impacted by social implications associated with stillbirth such as silence and stigma (4). Bereaved parents may experience negative psychological outcomes and increased risk of mental health conditions such as anxiety and depression (5, 6).

In the United States, primary consensus defines stillbirth as fetal death after 20 weeks gestation. If gestational age is unknown, stillbirth is defined as fetal weight greater than or equal to 350 grams, excluding pregnancy terminations (7). In the United States, approximately 21,000 babies, or 1 in 175 births, are impacted by stillbirth each year (8) and fetal autopsy is conducted in approximately 21% of stillbirths (9). The World Health Organization (WHO) classifies a baby who dies *in utero* after 28 weeks gestation as stillborn and reports that approximately 2 million stillbirths occur every year (10); however, the definition of stillbirth and rates of stillbirth vary widely among countries (11, 12). Additionally, across high- and upper-middle income countries, gaps persist in the lack of national strategies to prevent stillbirth, approaches to mitigate stigma surrounding stillbirth, and guidelines to provide bereavement care (11). Due to a lack of standardized protocols in evaluation and classification of stillbirth, low rates of perinatal autopsy (7, 9, 13), and silence and stigma associated with stillbirth (4), the study of specific causes of stillbirth and best practices for perinatal bereavement care is limited.

The frequency and type of perinatal bereavement care research have significantly increased in the past two decades, with the majority originating from the United States, United Kingdom, Australia, Canada and Sweden (14). Related qualitative research has notably increased over the past two decades (14), perhaps responding to the gap in valuing and understanding the lived experience of stillbirth. Qualitative literature has historically focused on the lived experience of mothers who have had a stillbirth; however, in recent years, a significant increase in the study of paternal perspectives and partner experiences of stillbirth has emerged [e.g. (15–20)].

Previous literature that has centered the lived experience of stillbirth ranges from specific interventions or memory-making activities (e.g. stillbirth photography) or post-loss outcomes (e.g. mental health conditions) to broader themes of the stillbirth experience. More often than not, the results from these qualitative studies have been directed to the healthcare workforce, being used to develop evidence-based clinical training and education (21, 22), support perinatal bereavement care policy (17), and identify post-hospital support services/strategies (18, 22). Notably, the development and updates of some national perinatal bereavement care guidelines incorporate bereaved parent perspectives, recognizing the significance of collaborating with those most affected by stillbirth and neonatal death (23, 24).

Minton et al. (3) revealed a gap in stillbirth literature: the lack of sharing firsthand recommendations about the stillbirth experience from parent-to-parent. Minton et al. (3) identified several overlapping themes with previous research, including the unique experience of grief and the grieving (18, 21), the importance of acknowledging the unique personhood of the baby (17, 22, 25), and validating the unique experience of motherhood (1). The authors’ focus was to share first-hand advice from stillbirth parents about post-loss coping strategies to support newly bereaved parents (3).

It is important and necessary that stillbirth research continues to enhance clinical practices to improve perinatal care for parents and families experiencing stillbirth; however, it is equally critical that newly bereaved stillbirth parents have accessible information and advice that addresses their post-loss needs, even more so from others who have shared a similar experience.

Most stillbirth research does not identify its audience as parents and/or families experiencing stillbirth, nor does it facilitate parent-to-parent advice, thus, making relevant recommendations unavailable or inaccessible to newly bereaved parents and families. This manuscript is part of a larger study surveying parents with lived experience of stillbirth about their advice to new parents experiencing loss. The goal of the larger study is to add to the limited peer reviewed literature documenting advice to newly bereaved parents for the development of support materials. There are many terms used to describe newly bereaved parents (26). Throughout the rest of this manuscript, in addition to the term “newly bereaved parents,” we also use the term “new loss parents” to describe parents who have very recently experienced or will soon experience a stillbirth as this is a colloquial term used by parents with lived experience of stillbirth. The aim of this study is to offer parent-to-parent advice to new loss parents experiencing a stillbirth to better prepare them for the birth of their stillborn baby. Specifically, this research brief asks advice from parents who have experienced a perinatal/fetal loss about “What should new loss parents know before the birth of their stillborn baby?”.

## Method

### Present study

A self-administered online survey using Qualtrics software (27) was used to gather the experiences from parents experiencing stillbirth or neonatal loss. While the survey asked respondents about several topics related to perinatal loss, only a subset of the data was included in this manuscript. Specifically, to be included in the data analysis for this manuscript, respondents must have fulfilled the inclusion criteria in **Table 1**.

**Table 1:** Inclusion criteria.

Inclusion Criteria
Be ≥18 years old
Have experienced a stillbirth (≥20 weeks gestation) OR have a self-identified stillbirth experience near 20-week gestation
Have access to the internet
Be fluent in written and verbal English
Consent to participate in the study
Answer the demographic questions and open ended question related to the research question

The content for the survey was developed based on previous literature on stillbirth [e.g. (18)] and neonatal loss [e.g. (6)] and authors’ experiences (the first author has personal experience with stillbirth, the second author has personal experience as a labor and delivery nurse). Survey design and questions were developed according to recommendations from Rea and Parker (28). For example, the question topics flowed in a logical order through the perinatal loss experience and questions asked about one topic at a time. Since parents experiencing stillbirth and neonatal loss often want their baby to be remembered (18), respondents were given the option of writing their baby’s name in a free response question to be used in upcoming survey questions and dissemination of the research findings. Respondents could also choose to not include their baby’s name or not have their baby’s name included in dissemination.

### Theoretical underpinnings

The two authors have different backgrounds related to stillbirth providing insider and outsider perspectives (29). The first author (JK) has the lived experience of stillbirth, providing an insider perspective on interpretation of participant responses. JK also has an outsider perspective as a researcher without any formal training in obstetrics. The second author (JL) has cared for patients and families of stillborn babies as a certified inpatient obstetric nurse, providing an insider perspective on interpretation of health care providers, systems, and processes. JL also has an outsider perspective as a parent who has not experienced a stillbirth.

### Ethics

A university Institutional Review Board (IRB) approved this study. The incentives for this project were funded by a small university grant. Respondents who completed the survey and met the inclusion criteria were eligible to be included in a raffle for one of 120 $5 gift cards or donations to an organization of their choice. Those who wished to be included in the raffle were asked to provide their name and contact information. Identifying information (other than the baby’s name) is known only to the two authors, is currently stored on a password protected computer, and will be deleted at the conclusion of the larger study. There was no formal patient/public involvement in this study; however, the authors have personal and/or professional experience related to stillbirth and parents with experience in stillbirth and/or neonatal loss piloted the survey.

### Data collection procedure

After IRB approval, a selected group of personal contacts who experienced either stillbirth or neonatal loss piloted the survey. The pilot group was asked for any feedback or suggestions for the survey. Minor edits were made to the survey based on this feedback (e.g., wording changes). Recruitment was multi-pronged and included outreach to personal contacts, posting on relevant social media pages, and contacting non-profit organizations supporting bereaved parents. Parents interested in completing the survey accessed the Qualtrics (27) link through fliers, posts, or emails to review the consent information. When clicking the Qualtrics (27) link, parents were immediately shown the consent for the study. If they consented to participate in the study, the survey began. If they declined to consent, the survey ended. Respondents could choose to skip any question unless it was required for inclusion criteria confirmation or data verification. Responses were anonymous, unless parents opted to include their baby’s name or opted to be included in a raffle.

Before data analysis, survey responses were rigorously scrutinized for respondent accuracy through a multi-step process including review of content, survey completion data, demographic information, and open-ended responses. For example, survey completion data such as start date/time, end date/time, and IP address were reviewed for duplicates. Any duplicate responses were reviewed in full and discarded if other questions were identical. The final analysis for this manuscript included 193 respondents who met the inclusion criteria and cleared the data cleaning process.

The majority of our respondents identified as white/Caucasian (79%), heterosexual (94%), females (94%) living in the USA (84%). Most participants had more than one pregnancy and 1–2 pregnancy losses, including a stillbirth which occurred between 1980-2025. The gestational age at first stillbirth ranged from 17-40+ weeks with the largest percentage between 33–40 weeks (57%). See **Table 2** for detailed demographic information and pregnancy history of the 193 parent respondents.

**Table 2:** Parent demographic data.

Item	n	%
Age	193	
20–29 years old	27	14.0
30–39 years old	106	54.9
40–49 years old	53	27.5
>50 years old	7	3.6
Gender	192	
Female	181	94.3
Male	10	5.2
Non-binary	1	0.5
Did not answer	1	0.5
Race/Ethnicity	193	
American Indian, Alaska Native, First Nations	1	0.5
Asian, Asian American	5	3.0
Black, African American	10	5.2
Hispanic	7	3.6
Latino/a/e	1	0.5
Middle Eastern, North African, Arab American	1	0.5
White, Caucasian	153	79.2
A race or ethnicity not listed here^a^	5	3.0
Multiple Race/Ethnicity^b^	10	5.2
Sexual Orientation	193	
Bisexual	7	3.6
Gay or lesbian	1	0.5
Heterosexual or straight	182	94.3
Prefer not to answer	2	1.0
An orientation not listed here	1	0.5
Residing Country	193	
USA	163	84.5
Other^c^	30	15.5
Parent Loss Experience	193	
Person who was pregnant	184	95.3
Partner	9	4.7
Number of Pregnancies	193	
1-3	134	69.4
4-6	54	30.0
7+	5	2.6
Number of Pregnancy Losses	193	
1-2	157	81.3
3-4	31	16.0
5+	5	2.6
Number of Stillbirths	193	
1	189	97.9
2	4	2.1
Gestational Age of First Stillbirth (weeks)	185	
17-24	37	20.0
25-32	35	18.9
33-40	106	57.3
>40	7	3.8
Year of First Stillbirth	192	
1980’s	1	0.5
1990’s	3	1.6
2000’s	10	5.2
2010’s	56	29.1
2020’s	122	63.5

*n* varies per item 185–193 as indicated for each item.

a A race or ethnicity not listed here included Australian n=2, Irish n=1, Sri Lankan n=1, and French n=1.

b Multiple Race/Ethnicity respondents included Latino/a/e, Brazilian n=1; Black African, Portuguese n=1; Hispanic, White, Caucasian n=2; Black, African American, White, Caucasian n=2; Hispanic, Latino/a/e n=1; and Hispanic, Latino/a/e, White, Caucasian n=1.

c Other (residing countries with <10 respondents) were Canada n=8, UK n=7, Australia n=5, South Africa n=2, Denmark n=1, Germany n=1, Ireland n=1, Netherlands n=1, Rwanda n=1, Uzbekistan n=1, United Arab Emirates n=1, and Venezuela n=1.

### Data analysis and reliability

Data analysis for this manuscript included two parts. First, demographic questions were analyzed using frequency counts and percentages. Second, the open ended question was analyzed using content analysis. The first and second author reviewed all responses to the question, “What should new loss parents know before the birth of their stillborn baby?” then met to discuss possible clusters. The first and second author generated operational definitions of clusters and applied them to a subset of the data. After revising the clusters and operational definitions, the first and second author coded 36% of the data to obtain inter-rater reliability. Inter-rater reliability was calculated by dividing the number of correct (matching) codes by the total number of codes. Inter-rater reliability for these data was 84% which is considered reliable and not likely to occur by chance (30). Once inter-rater reliability reached at least 80%, the first author coded the remainder of the data. Any discrepancies were discussed by both authors until there was agreement. This content analysis generated five clusters; however, only clustersthat included at least 20% of participants were included in this manuscript.

## Results

Parent participants discussed a variety of topics they felt were important with varying lengths of detail ranging from a few words to a few paragraphs. Content analysis generated four main clusters respondents felt new loss parents should know before the birth of their stillborn baby: (1) the birthing process, (2) the decisions about their baby, (3) memory making activities, and (4) the emotions before and after their baby’s birth. See **Table 3** for additional example quotes.

**Table 3:** Summary of clusters and codes for what new parents should know before the birth of their stillborn baby.

Cluster	Code	Examples of code	% (n)
Birthing process			37% (72)
	During birth	*Birthing Process* ”You are still going to experience everything.” - Auden’s mother ”You still have to give birth.” - Jayden’s mother ”Have a clear understanding of how the process will work” *Advocacy* ”You are in charge. Do not let anyone set the place for the day ahead of you.” - Karter’s mother ”State your wishes with the hospital clearly.” - Patrick’s mother *Environment* ”Your delivery will most likely be just like any other, minus the cries” - Alliyah’s mother ”It’s quiet” - Max’s mother	35% (67)
	After birth	*Appearance* ”Your baby may have some changes that have happened to their body since passing away that you will see when they are delivered.” - Julian’s mother ”I would like to have had more information about how my baby’s body would be/look/deteriorate after he was born.” - Finn’s mother *Postpartum* ”It sounds obvious, but you still have to go through the exact same birth and postpartum process as with a live baby.” - Brynn’s mother ”Your milk might still come in, it sucks” - Luke’s mother	11% (22)
Decisions about their baby			23% (44)
	Options for memory making	“Someone will ask you if you want to hold your baby, what you want to name them, if you want birth or newborn pictures taken.” – Lucas’ mother ”Have a plan for after baby is born. Example: do you want to hold baby right away?” “They should do the things they had in mind to do with their baby anyway.” – Liam’s mother	14% (27)
	Options for post-birth care	*Memorializing* ”Also know you will have to make decisions with a funeral home, if you would like a service, burial, or cremation.” - Carter’s mother ”Talk about what you/and your partner wishes to do with your stillborn child. Burial, cremation, remains buried or in an urn. It is also okay to change your mind after, but it will be harder to decide, once you’ve delivered.” - Hugo’s mother *Medical Decisions* ”Request genetic testing inclusive of whole genome sequencing at delivery, request full autopsy with placental pathology” - Oliver’s mother ”And if you want answers, you can always have an autopsy.” - Finnley’s mother	13% (25)
Activities of memory making			54% (105)
	Photo/Video	“Ask hospital staff to take pictures/videos, if even at the time you won’t feel like it - doesn’t matter if you look at them later at all, important thing is you have them if you ever want to.” - Hannah’s mother ”Take a picture of them with you” - Charle’s mother	36% (70)
	Parenting	“Sing and bathe and clothe them” - Linclon’s mother ”Read to them” - Lily’s mother	26% (51)
	Time/Memories	“Spend as much time with your baby as you can” – Ellie’s mother ”Make as many memories as possible” - Madison’s mother	25% (48)
	Physical Contact	“Hold your child” - Ford’s mother ”Cuddle them” - Theodore’s father ”Kiss them” - Lukas’s mother	18% (35)
	Physical Memento	“Get hand prints and foot prints” - Auden’s mother ”Snip a locket of hair” - Mara’s mother ”Keep an outfit” - Elaina’s mother	12% (24)
	Family	“Invite your loved ones to meet your baby” - Thomas’s mother ”Include your older children, no matter their age, especially if they knew that you were expecting … let your children hold their sibling.” - Simeon’s mother	5% (10)
	Viewing	“Look at their toes/fingers, try to remember all tiny details” - Hannah’s mother ”You might not want see your baby but when you do you will see just how amazing they look” - Miracle’s mother	4% (8)
Emotions before and after their baby's birth			48% (93)
	Emotions in the moment	“This is one of the hardest things they will go through in their life.” - Noah’s mother ”You are going to be devastated more than you could ever prepare yourself for.” - Ryder’s mother “I know this can be challenging in the moment, because no mom expects their baby to die. But once you receive this news, not only will you be experiencing a variety of emotions, your mind will be racing about what is to come next.” - Garth’s mother ”Nothing can prepare you for the rollercoaster of feelings. Delivery is going to be slow and fast at the same time. You will want it to just be over and then to have it all back.” “So many emotions will be experienced, both you and your partner will go through them and it’s ok.” - Benjamin’s mother	30% (57)
	Emotions in the future	“Those moments after will never be forgotten.” - Rosalia’s mother ”It is something you will never understand, never come to terms with, never forget. Soak up every moment you get to spend with your baby.” - Charlee’s mother ”Only another loss parent can really understand. It will completely change who you are. You don’t get over it, you learn to live with it. It is not the end - there is life after loss.” - Aubrey’s mother ”You need to grieve and talk about it. There will be people who you thought would be there, that won’t. This experience is unforgettable and will change you as a person. Take what you have experienced and help others who are going through the same thing.” - Dylan’s mother	23% (44)
	Emotions of self-perception	“There is no right or wrong way to navigate loss. Anything you want, feel is validated without explanation.” - Meredith’s mother ”It is not your fault. Your child has known nothing but love and comfort from you.” - Callie’s mother ”You aren’t alone. Don’t be hard on yourself. It’s okay to smile or laugh. It is not your fault.” - Liam’s mother	26% (39)

Individual participant responses could be coded as multiple clusters and codes.

Many of the 193 responses (54%) had statements that contained multiple clusterswith the most common pairing consisting of comments related to activities of memory making and emotions experienced before and after their baby’s birth. For example, parent participants frequently commented about participating in a memory making activity with their baby and how it may be difficult, but it is time you only get once. A response from Thomas’s mother summarized this concept well:

“This isn’t your fault. You didn’t do anything for your baby to die. You can do this. You can give birth to your child and hold them. This is going to be the only time physically here on earth that you can have with them, cherish it. Even though it’s hard, don’t be afraid to hold them and look at their faces. Take pictures, videos, invite your loved ones to meet you baby. They’re still your baby and all they felt in their life was warmth and love.”.

### The birthing process

Approximately 37% of open-ended responses included the cluster of knowing about the birthing process. These were comments related to two main experiences: during birth and after birth. Respondents discussed three main codes related to the birthing process of their stillborn baby: information about the birthing process, the hospital environment, and self-advocacy. The most common code focused on new loss parents needing information about the birthing process for a stillborn baby. Parent participants mentioned that even though their baby no longer had a heartbeat, the labor is similar. For example, parent participants commented “you still have to give birth” (Jayden’s mother) and “you are still going through the process of labor and seeing your precious baby … even if it’s not the way you expected.” (Elliana’s mother). A few respondents normalized the process of understanding what a birthing experience with a stillborn baby would be like. One parent commented, “This seems obvious but it actually hit me that in the midst of all of this sadness, you still have to give birth.” (Lily’s mother).

As part of understanding the birthing process, parent participants also commented on the environment surrounding the delivery of a stillborn baby. Many of these comments were related to the silence that “will be deafening” (Emberly’s mother) or trying to prepare new loss parents for the sounds they would likely hear such as “you will hear other women giving birth, babies crying.” (Eden and Elena’s mother). Respondents also encouraged new loss parents to advocate for themselves and their needs during pregnancy (e.g., “Don’t ignore the signs. If you feel weird and get dismissed, still get checked.” - Asher’s mother) and at the hospital (e.g., “Ask all questions you need, even if you need to ask a few times. That time is a massive blur and can be hard to take things in.” - Meadow’s mother).

There were two codes related to the experience after birth. First, parent participants commented on how a stillborn baby’s appearance changes after birth and how they were not prepared for these changes. Parent participants gave varying levels of detail in warning new loss parents about their baby’s appearance changes after birth, with some participants offering general descriptions (e.g., “your baby may have some changes that have happened to their body since passing away that you will see when they are delivered” - Julian’s mother) while others offering more specific details (e.g., “they will probably have skin peeling, discoloration, bruising and bleeding” - Lincoln’s mother). Many respondents from this code commented they were not prepared for how their baby’s appearance would be different from a living newborn or how their baby’s appearance would change over time. Charlie’s mother said, “Charlie was born with skin slippage. I wasn’t prepared for that.”.

Second, parent respondents highlighted that all of the typical postpartum changes still occur, even without a living baby. These postpartum changes were often discussed in relation to lactation. For example, one parent said, “Your milk will come in after a stillbirth and it will be very painful, both physically and emotionally. No one prepared me for this.” (Mia’s mother).

### The decisions about their baby

Approximately 23% of parent participants (44) commented about decisions new loss parents would need to make about their stillborn baby after delivery. Comments related to this cluster were coded as options for memory making and options for post-birth care. Respondents described options for memory making as either hypotheticals for new loss parents to consider or information for healthcare professionals to provide. For example, Madelyn’s mother said, “…think about how you want your birth or time after to be. Do you want to hold him/her? Do you want alone time? Do you still want skin to skin?” Other parent participants encouraged options to be given to the new loss parents so they can use their limited amount of time purposefully. Madison’s mother said, “I feel like they should be told in as gentle a way as possible what to expect and the range of options available to them once their baby is born.” Some parent participants expressed they would have liked more information about what other loss parents had done because they didn’t know what the options were, but they “wish[ed] someone could have talked me through what others do to normalize it all.” (Finn’s mother).

Other comments about decisions new parents would need to make about their stillborn baby were related to post-birth care. First, parent participants commented about deciding how to memorialize their baby such as cremation or burial. Some respondents acknowledged the difficulty in making these decisions such as one parent who commented, “There are things like funeral and burial that you never thought you would have to think about.” Second, parent participants mentioned the need to make medical decisions about their baby such as whether or not to have genetic testing, an autopsy, or placental pathology. One parent commented, “A perinatal autopsy should be encouraged for a cause of death and knowledge for subsequent pregnancies.” (Valentina’s father).

### The activities of memory making

Over half of the participants (54%) advised new loss parents to do at least one memory making activity. In fact, 61% of respondents recommended new loss parents do at least two (range 1-6, average 2) memory making activities with their stillborn baby. The most common pairings were taking photos/videos combined with having physical contact (27), spending time with their baby (24), or doing a parenting activity (21). Some advice to new loss parents was provided as a list of options such as, “Do all the memory-making options (foot molds, lock of hair, etc.).” (Kaia’s mother). Other advice was more detailed to the specific type of memory making activity such as the parent who commented:

“I wish someone would have told me to take all the pictures you can, spend as much time as you can with your baby, and even though it’s such as painful time, to make the most out of the time you have with your baby. I always tell loss parents this now.” (Rorie’s mother).

### The emotions before and after their baby’s birth

Approximately 48% of parent participants (92) offered advice to new loss parents related to the emotions before and after their baby’s birth. Comments in this cluster were coded as emotions in the moment, emotions in the future, and emotions of self-perception. Many of these parent participants (41%) offered advice in more than one code. The most frequent combination was discussing the emotions in the moment and emotions in the future. Respondents commented that in the moment experiencing the birth of your stillborn baby is “one of the hardest things you ever do” and is “one of the worst days of your life,” but that it is also important to “be present” (Vincent’s mother) and “take the time to make memories” (Jules’ mother) because “they are still your child and you are still their parent” (Emma’s mother) and “you will cherish those memories forever.” (Elliana’s mother). For example, Gus’ mother said:

“I think they should know that they can spend time with their baby and take photos and try to create some memories. This may seem scary or they may feel like they don’t want to do this but I truly believe as time passes they will wish that they had embraced every moment that they get with their child.”.

Parent participants often described emotions in the moment at the hospital for new loss parents giving birth to their stillborn baby as overwhelming, painful, and difficult. Several parent participants commented in a variety of ways that the birth of their stillborn baby is “truly a devastating time” (Gabriel’s mother) and is “the absolute worst thing you will ever go through” (Lila’s mother). Some respondents offered encouragement to new loss parents by adding that they can do this, they will survive this, and there is support for them. Respondents also emphasized new loss parents need to “give themselves grace” (Jules’ father) for the decisions they need to make in the hospital. Additionally, parents addressed emotions of self-perception, or feelings new parents may have about themselves, by offering comfort to new loss parents letting them know “they are not alone” (John’s father), and telling them what happened was “not their fault” (Blakelyn’s mother). For example, Malakhi’s mother wrote:

“Remember that this is not your fault. Guilt hits hard at the beginning because we might feel we are responsible for our baby’s health. However, there are things out of our control and stillbirth is one of them.”.

Finally, respondents provided advice and guidance for emotions in the future the new loss parents may feel as time passes since the birth of their stillborn baby. Respondents indicated that “grief takes time” (Destiny’s mother) and the “journey is difficult.” Several parent participants commented on the lasting, but changing nature of grief, highlighting that your baby being stillborn “will change you forever” (Jasper’s mother) and grief is something “you don’t get over … you learn to live with it” (Aubrey’s mother). One reason respondents gave for this lasting grief that changes the parent is that “you are still a mom/dad/parent, even when your baby dies” (Rory’s mother).

## Discussion

### Summary of main findings

While there is peer reviewed literature of parents describing their stillbirth experiences [e.g. (18)], there are few from the perspective of offering parent-to-parent advice. This manuscript builds on Minton et al. (3) and Conroy et al. (31) by focusing on advice parents who experienced the birth of a stillborn baby would give to new loss parents experiencing stillbirth before going to the hospital. Newly bereaved parents who recently found out their baby would be stillborn would likely benefit from guidance from someone who understands their situation, just as other bereaved parent populations have benefited [(e.g., childhood cancer (32)]. Analysis of peer-to-peer support programs for bereaved parents whose children died from childhood cancer noted a variety of different support parents offered to newly bereaved parents (32). Similarly, someone with the lived experience of stillbirth is able to reflect on the most important topics for new loss parents to know and provide resources, strategies, or other practical tips for different stages of the grief journey. Information parent participants in this study felt new loss parents should know were categorized into four main clusters: the birthing process, the decisions about their baby, the memory making activities, and the emotions before and after their baby’s birth.

### Interpretation

Statements from parent participants combining the two clusters of memory making activities and emotions before and after their baby’s birth were some of the most frequent clusters throughout the data set. This frequency highlights the importance of making memories at the hospital for the future self, but also validates the difficulty of these actions in the moment. Parents in this study suggested a wide range of memory making activities such as taking photos or videos, doing parenting activities (e.g., bathing), having physical contact (e.g., holding) and many general statements encouraging new parents to make as many memories as they can and spend as much time with their baby as possible. Similarly, best practice guidelines (7, 23, 24) and previous literature (5) encourage memory making activities including spending time with the baby, creating mementos, and seeing or holding the baby (33). Some guidelines recommend that healthcare professionals repeatedly offer a variety of memory-making opportunities to parents (23, 34), but encouragement alone may not be sufficient., It may be important to couple suggestions for memory making activities with normalizing the complex emotions parents reported feeling in this study. Parents have suggested healthcare providers address the emotional component of taking photographs to reduce barriers specifically related to postmortem photos (35), but this may be a helpful strategy for all memory making activities.

Approximately 37% of respondents provided advice related to the birthing process with most advice centering on the birth experience such as going through the typical birthing process, advocating for what the parent needs, and the birthing environment. Many parent participants pointed out that even though your baby died, the birthing process is the same. Anecdotally, the concept of still giving birth after finding out your baby no longer has a heartbeat is a common shock reported by parents who experienced stillbirth describing their experiences in podcasts [e.g. (36, 37)]. A smaller percentage of respondents provided advice for after the birth of the stillborn baby related to the baby’s appearance after birth and postpartum physical changes. his advice seems critically important because several parents also commented they were not informed of physical changes of their baby or the postpartum period during their stillbirth experience. Parents interviewed in Nuzum et al. (25), also briefly mentioned being worried about what their baby may look like. The baby’s changing appearance in moments and hours after birth may be important to communicate to parents.

Nearly a quarter of respondents reflected on the time-sensitive and unexpected decisions that they needed to make about their stillborn baby following delivery. Based on parent respondent comments, new loss parents experience complex emotions they must navigate while also being asked to make difficult decisions related to the immediate and post-mortem care of their stillborn baby, some of which may impact future pregnancies. Informed and shared decision making is a key characteristic of perinatal bereavement care guidelines (14, 23, 24, 34). Healthcare providers can implement shared decision making by giving parents accurate information about the options available to them and supporting the decisions they make that shape their experience (14). As with memory-making activities, newly bereaved parents may not know or may not know how to ask about options surrounding post-birth care of their stillborn baby. If new loss parents are aware of the options from which they can choose, they may feel more agency in the decisions they are making during a difficult time.

Parent-to-parent advice about the emotions before and after the birth of the baby addressed emotions in the moments after the baby is born, emotions related to the parents and emotions in the future after leaving the hospital and beyond. Advice from this cluster reassured new loss parents that their baby being stillborn was not their fault, validated the difficulty of the moment, and emphasized the enduring grief in the future. The most common advice offered by parent participants in this cluster was a combination of emotions in the moment and emotions in the future. Parent participants validated the current pain of the new loss parents after their stillborn baby was born and the lasting pain after leaving the hospital as they process their grief. It is important for newly bereaved parents to understand that their experience is unique and their grieving process is not linear, nor does it have a set timeline (3). Parent reports of their grief experiences align with many grief theories, such as the integrated process model, which also describe grief as multifaceted, interconnected, and individualized (38). One method of coping with grief after stillbirth is to attend support groups and/or connect with peer-to-peer support to find community through a shared experience and validate parenthood (3, 21, 31).

Various national perinatal bereavement care guidelines outline evidence-based and consensus-based recommendations for the care of patients and families experiencing perinatal loss (23, 24, 34). While bereaved parent perspectives may have been considered in their development, these guidelines are specifically directed toward the interdisciplinary healthcare professionals who provide perinatal care. The results from the current study reflect many from previous stillbirth literature and current perinatal bereavement care recommendations; however, they are directed to peers who have the lived experience of stillbirth.

### Strengths, limitations, and future directions

Strengths for this study include the research team, composed of individuals from insider and outsider experiences of stillbirth and healthcare, and the breadth of experiences discussed in the data. The number of respondents (N = 200) provided a range of advice in different areas, varying in length from a phrase to several sentences. While these responses represent a small fraction of the annual stillbirths in the USA and around the world, the data were rich descriptions. Additionally, the sample was fairly homogenous demographically with the majority of respondents identifying as white, heterosexual females living in the USA. Future research should examine the experiences and advice from individuals representing a variety of demographic categories.

## Conclusion

The parent-to-parent advice from this manuscript may serve as a guide for parents experiencing stillbirth, health care and mental health professionals, and organizations supporting bereaved parents and families. Parents who recently found out their baby would be stillborn may benefit from the advice in this manuscript by being more prepared for their hospital and birth experience. For example, they may feel more comfortable asking questions, making memories with their baby, or allowing themselves to feel a variety of emotions. Professionals may benefit from the advice in this manuscript by better understanding the patient perspective during the hospital stay without relying solely on the patient who may or may not know what they need or what to ask in this situation. For example, healthcare professionals may not realize the parents want to know what their baby will look like, but don’t know how to ask or if they can ask. Mental health professionals may use the advice to help newly bereaved parents overcome their guilt related to their baby’s death or cope with how losing their baby will change them. Organizations supporting parents and families may use this advice to guide peer-to-peer training for support groups or parent mentors and parent resources. Many organizations supporting parents and families who experience a stillbirth have volunteers who have lived experience; however, having the additional support from multiple experiences backed by research literature may improve training and/or mentoring.

## Data Availability

The raw data supporting the conclusions of this article will be made available by the authors, without undue reservation.
